# Neutralization of Omicron BA.1, BA.5.1.6, BQ.1.3 and XBB1.1 induced by heterologous vaccination Ad5-nCoV and mRNA-1273

**DOI:** 10.1038/s41392-023-01447-y

**Published:** 2023-04-29

**Authors:** Jesús Hernández, Freddy Dehesa-Canseco, Alma B. Vázquez-López, Mónica Reséndiz-Sandoval, Graciela Caire-Juvera, Mario Solís-Hernández, Olivia Valenzuela, Bruno Gómez-Gil, Verónica Mata-Haro

**Affiliations:** 1 Centro de Investigación en Alimentación y Desarrollo, A.C., Hermosillo, Sonora 83304 Mexico; 2 Comisión México-Estados Unidos para la Prevención de la Fiebre Aftosa y otras Enfermedades Exóticas de los Animales (CPA), SENASICA, SADER, Ciudad de México, 05010 Mexico; 3 Departamento de Ciencias Químico Biológicas, Universidad de Sonora, Hermosillo, Sonora 83000 Mexico

**Keywords:** Infectious diseases, Vaccines

**Dear Editor**,

The emergence of Omicron subvariants has required additional boosters to induce a highly neutralizing antibody response.^1^ However, the new Omicron subvariants BQ.1 and XBB, evade the humoral response induced by multiple mRNA vaccinations and infections.^2,3^ CansinoBio (Ad5-nCoV) is a single-dose adenovirus vector-based vaccine with high protection against severe disease^4^ but lower efficacy and antibody response than mRNA vaccines. Fortunately, hybrid immunity induced by infection plus vaccine or heterologous vaccines has produced a robust neutralizing antibody response.^5^

In this work, we evaluated neutralizing antibodies against the SARS-CoV-2 ancestral strain (B.1.189) and Omicron BA.1, BA.5.1.6, BQ.1.3, and XBB.1 subvariants in serum samples from nonhospitalized adult participants immunized with a single dose of Ad5-nCoV and a booster eight months later with the mRNA-1273 vaccine. Priming with Ad5-nCoV was administered by May 2021 as part of Mexico’s National COVID-19 vaccine program. In January 2022, the subjects received the booster dose. Blood samples were collected at baseline (before the booster, *n* = 314) and three weeks after the mRNA-1273 booster (*n* = 188). The Ethics Committee of CIAD evaluated and approved this study (CEI/005-2/2020), and all patients signed informed consent forms. We used ELISA to analyze the anti-N and anti-RBD antibodies and a microneutralization assay with live virus to evaluate neutralizing antibodies against the ancestral strain and the Omicron BA.1, BA.5.1.6, BQ.1.3, and XBB.1 subvariants. All the samples were collected in Hermosillo, Sonora, Mexico. Demographic data are summarized in supplementary Table 1.

First, we evaluated antibody persistence in individuals after eight months of one dose of the Ad5-nCoV vaccine. Approximately 70.38% (221/314) of the samples showed detectable neutralizing antibodies (nAbs) (titer > 1:10) against the ancestral strain, 61.10% (195/314) against BA.1, 58.91% (185/314) against BA.5.1.6, 4.45% (14/314) against BQ.1.3, and 4.77% (15/314) against XBB.1. The geometric mean neutralizing titers (GMTs) were 27.70, 14.20, 12.28, 3.25, and 3.17 for the ancestral strain, BA.1, BA.5.1.6, BQ.1.3 and XBB.1 subvariants, respectively. These values represent 1.95-, 2.25-, 8.52-, and 8.73-fold reductions in BA1, BA.5.1.6, BQ.1.3, and XBB.1, respectively, compared with the ancestral strain (Fig. 1a). Next, we classified individuals by their SARS-CoV-2 infection status: previously infected, 58.82% (169/314) and uninfected, 46.17% (145/314) (supplementary Fig 1). The GMTs in the previously infected group were 60.18, 26.76, 22.16, 3.75, and 3.69 against the ancestral strain, BA.1, BA.5.1.6, BQ.1.3, and XBB.1, respectively, with reduction folds of 2.24, 2.71, 16.04, and 16.30 for BA.1, BA.5.1.6, BQ.1.3, and XBB.1, respectively, compared with the ancestral strain. In the nonpreviously infected group, the GMTs were 11.11, 6.72, 6.17, 2.68, and 2.66 against the ancestral strain, BA.1, BA.5.1.6, BQ.1.3, and XBB.1 subvariants, respectively, with fold reductions of 1.65, 1.80, 4.14, and 4.17 for BA.1 BA.5.1.6, BQ.1.3, and XBB.1, respectively, compared with the ancestral strain.

**Fig. 1:**
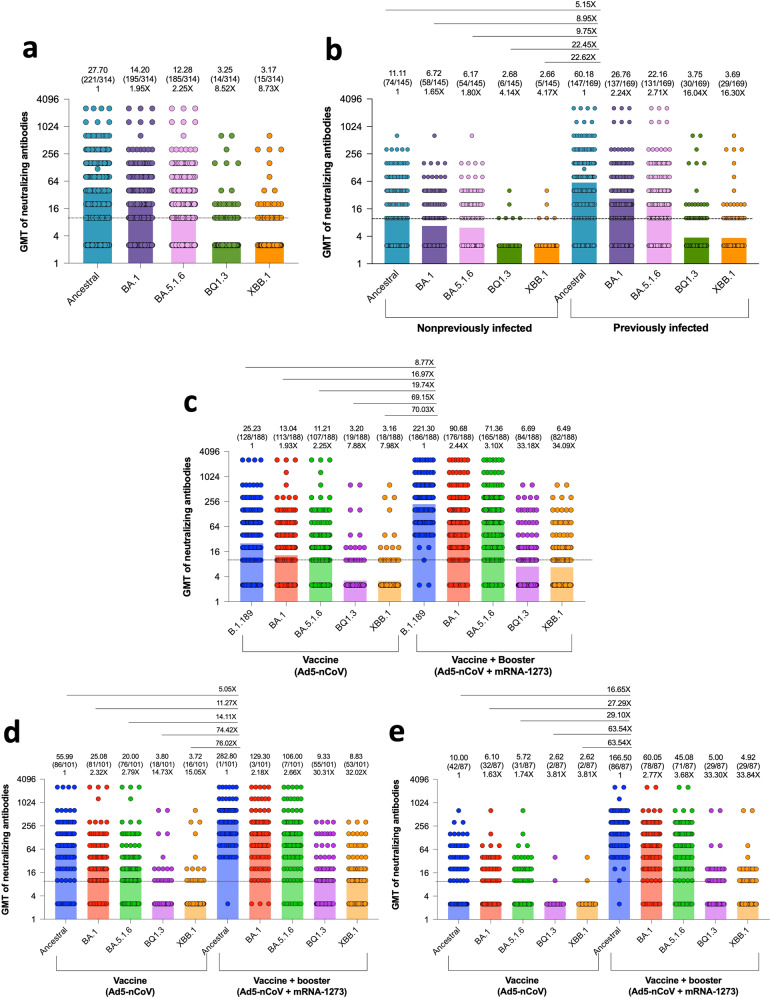
Neutralizing response against the ancestral strain and Omicron BA.1, BA.5.1.6, BQ.1.3, and XBB.1 subvariants induced by Ad5-nCoV and a heterologous immunization of Ad5-nCoV and mRNA-1273. **a** Neutralizing antibodies (nAbs) against the ancestral strain and BA.1, BA.5.1.6, BQ.1.3, and XBB.1 of samples from individuals vaccinated with Ad5-nCoV. The *p* values (two-sided) for group comparison of GMT are as follows: ancestral strain versus BA.1, BA.5.1.6, BQ.1.3, and XBB.1: all <0.0001. **b** nAbs against the ancestral strain and Omicron BA.1, BA.5.1.6, BQ.1.3, and XBB.1 subvariants of samples from individuals nonpreviously infected and previously infected vaccinated with Ad5-nCoV. The *p* values (two-sided) for group comparison of GMT are as follows: ancestral strain versus BA.1, BA.5.1.6, BQ.1.3, and XBB.1 (in both nonpreviously infected and previously infected): all <0.0001. **c** nAbs against the an**c**estral strain and BA.1, BA.5.1.6, BQ.1.3, and XBB.1 of samples from individuals at baseline and after the booster; the p values (two-sided) for group comparison of GMT are the following: ancestral strain versus BA.1, BA.5.1.6, BQ.1.3, and XBB.1 (in both vaccine and vaccine + booster): all <0.0001), and (**d**) samples from individuals previously infected; the *p* values (two-sided) for group comparison of GMT are the following: ancestral strain versus BA.1, BA.5.1.6, BQ.1.3 and XBB.1 (in both vaccine and vaccine + booster): all < 0.0001) and (**e**) nonpreviously infected; the *p* values (two-sided) for group comparison of GMT are the following: ancestral strain versus BA.1, BA.5.1.6, BQ.1.3 and XBB.1 (in both vaccine and vaccine + booster): all <0.0001). From all figures, values above the bars denote geometric mean titers (top), the number in the parentheses indicates the proportion of positive samples with nAbs above the limit of detection (dotted lines ≥ 1:10) (middle), and values with an “X” indicate fold reduction (bottom). Statistical significance was determined by two-tailed Wilcoxon matched-pairs signed-ranks tests or Mann‒Whitney tests, and *p* values less than 0.05 were considered statistically significant

Then, we analyzed the booster effects of the mRNA-1273 vaccine in individuals primed with a single dose of the Ad5-nCoV vaccine. The IgG reactivity against the RBD was 85.10% (160/188) at baseline and 100% (188/188) after the booster (supplementary Fig. 2). The GMTs of nAbs at baseline were 25.23, 13.04, 11.21, 3.20, and 3.16 against the ancestral strain and BA.1, BA.5.1.6, BQ.1.3 and XBB.1, respectively, with fold reductions of 1.93, 2.25, 7.88 and 7.98 for BA.1, BA.5.1.6, BQ.1.3 and XBB.1 when compared with the ancestral strain (Fig. 1c). The GMTs after the booster were 221.30, 90.68, 71.36, 6.69, and 6.49 against the ancestral strain and BA.1, BA.5.1.6, BQ.1.3, and XBB.1, with reduction folds of 2.44, 3.10, 33.18, and 34.09 for BA.1, BA.5.1.6, BQ.1.3, and XBB.1 when compared with the ancestral strain. The GMTs fold reduction for BA.1, BA.5.1.6, BQ.1.3, and XBB.1 in the group before the booster versus the ancestral strain of the group after the booster was 16.98, 19.74, 69.15, and 70.03, respectively (Fig. 1c). These results showed that BQ.1.3 and XBB.1 have a significant capacity to evade the nAbs induced by the combination of Ad5-nCoV and the mRNA-1273 vaccine.

Then, the individuals were grouped according to their COVID-19 status, previously infected and nonpreviously infected. In each group, we compared the GMTs before and after the booster. In the previously infected group (Fig. 1d), 85.14% (86/101), 80.19% (81/101), 75.24% (76/101), 17.82% (18/101), and 15.84% (16/101) showed nAbs against the ancestral strain and BA.1, BA.5.1.6, BQ.1.3, and XBB.1, respectively, before the booster. The GMTs against the ancestral strain and BA.1, BA.5.1.6, BQ.1.3, and XBB.1 subvariants were 55.99, 25.08, 20.00, 3.80, and 3.72 with reduction folds of 2.32, 2.79, 14.73, and 15.05 for BA.1, BA.5.1.6, BQ.1.3, and XBB.1 subvariants when compared with the ancestral strain. After the booster, 99.00% (100/101), 97.02% (98/101), 93.06% (94/101), 54.45% (55/101), and 52.47% (53/101) showed nAbs against the ancestral strain and BA.1, BA.5.1.6, BQ.1.3, and XBB.1 subvariants, respectively. The GMTs against the ancestral strain and BA.1, BA.5.1.6, BQ.1.3, and XBB.1 subvariants were 282.80, 129.00, 106.00, 9.33, and 8.83 with reduction folds of 2.18, 2.66, 30.31, and 32.02 for BA.1, BA.5.1.6, BQ.1.3, and XBB.1 subvariants, respectively, when compared with the ancestral strain. When compared to the GMTs before and after the booster, in the group of individuals previously infected, the fold changes were 5.05, 11.27, 14.11, 74.42, and 76.02 for the ancestral strain, BA.1, BA.5.1.6, BQ.1.3, and XBB.1, respectively. In the nonpreviously infected group before the booster (Fig. 1e), 48.27% (42/87), 36.78% (32/87), 35.63% (31/87), 2.29% (2/87), and 2.29% (2/87) showed nAbs against the ancestral strain and BA.1, BA.5.1.6, BQ.1.3, and XBB.1, respectively. After the booster, 98.85% (86/87), 89.65% (78/87), 81.60% (71/87), 33.33% (29/87), and 33.33% (29/87) showed nAbs against the ancestral strain and BA.1, BA.5.1.6, BQ.1.3, and XBB.1, respectively. The GMTs in the nonpreviously infected group before the booster were 10.10, 6.10, 5.72, 2.62, and 2.62 for the ancestral strain and BA.1, BA.5.1.6, BQ.1.3, and XBB.1, respectively, and 1.63-, 1.74-, 3.81-, and 3.1-fold reduction compared with BA.1, BA.5.1.6, BQ.1.3, and XBB.1, respectively. In contrast, the GMTs after the booster were 166.50, 60.05, 45.08, 5.00, and 4.92 for the ancestral strain and BA.1, BA.5.1.6, BQ.1.3, and XBB.1, respectively, and 2.77-, 3.68-, 33.30- and 33.84-fold reductions compared with BA.1, BA.5.1.6, BQ.1.3, and XBB.1, respectively. When comparing the GMTs before and after the booster, in the group of individuals nonpreviously infected, the fold changes were 16.65, 27.29, 29.10, 63.54, and 63.54 for the ancestral strain, BA.1, BA.5.1.6, BQ.1.3, and XBB.1, respectively.

Limitations of our study. First, we did not know the exact time of infection since the participants only reported a COVID-19 infection before or after the Ad5-nCoV vaccine. Second, we could not obtain the same number of samples at baseline and after the booster; hence, the analysis after the booster was performed with a lower number of samples.

In conclusion, these results demonstrate that nAbs induced by the Ad5-nCoV vaccine can persist after eight months, similar to other COVID-19 vaccines.^6^ However, the nAbs against Omicron BA.1 and BA.5.1.6 were lower and negative against BQ.1.3 and XBB.1. A heterologous booster with the mRNA-1273 vaccine increases nAbs, especially in previously infected individuals. However, the nAbs against BQ.1.3 and XBB.1 were still low, showing that vaccines, boosters, and infection are insufficient to neutralize these subvariants robustly. Assuming that a low neutralization response against the BQ.1 and XBB subvariants was observed even with four doses of mRNA vaccine^2^ or BA.5 bivalent booster,^7^ the combination of Ad5-nCoV vaccine with mRNA vaccines can be recommended as an immunization protocol against COVID-19.

## Supplementary information

SUPPLEMENTAL MATERIAL

## Data Availability

The lead contact will share data reported in this paper upon reasonable request.
