# Supplementary material for: Neutralization of Omicron BA.1, BA.5.1.6, BQ.1.3 and XBB1.1 induced by heterologous vaccination Ad5-nCoV and mRNA-1273
Source: Signal Transduct Target Ther. 2023 Apr 29;8:174. doi: 10.1038/s41392-023-01447-y (PMC10147998; doi:10.1038/s41392-023-01447-y)
Supplement: Supplementary file 1 — SUPPLEMENTAL MATERIAL [file 41392_2023_1447_MOESM1_ESM.docx]

Supplementary Materials for

**Neutralization of Omicron BA.1, BA.5.1.6, BQ.1.3 and XBB1.1 induced by heterologous vaccination Ad5-nCoV and mRNA-1273**

Jesús Hernández^1*^, Freddy Dehesa-Canseco^2^, Alma B Vázquez-López^1^, Mónica Reséndiz-Sandoval^1^, Graciela Caire-Juvera^1^, Mario Solís-Hernández^2^, Olivia Valenzuela^3^, Bruno Gómez-Gil^1^, Verónica Mata-Haro^1^

^1^ Centro de Investigación en Aimentación y Desarrollo, A.C., Hermosillo, Sonora, 83304 and Mazatlán Sinaloa 82000, Mexico.

^2^ Comisión México-Estados Unidos para la Prevención de la Fiebre Aftosa y otras Enfermedades Exóticas de los Animales (CPA), SENASICA, SADER, Ciudad de México 05010, Mexico.

^3^ Departamento de Ciencias Químico Biológicas, Universidad de Sonora, Hermosillo, Sonora, 83000, Mexico.

*Correspondence to: Jesús Hernández ([jhdez@ciad.mx](mailto:jhdez@ciad.mx))

**Materials and Methods**

Patient information and sample collection

All individuals in this study received one dose of Ad5-nCoV and a booster with the mRNA-1273 vaccine as part of Mexico’s National COVID-19 vaccine program. The Ad5-nCoV vaccine was administered by May 2021, and a booster was administered in January 2022 eight months later. Blood samples were collected at baseline (before the booster, n=314) and three weeks after the mRNA-1273 booster; unfortunately, only 194 samples were collected after the booster. All the samples were collected in Hermosillo, Sonora, Mexico. All participants answered a demographic survey and were further divided into previously infected and nonpreviously infected groups. All patients signed informed consent forms, and the study was evaluated and approved by the Ethics Committee of the CIAD, AC (CEI/012-2/2020).

Microneutralization

Vero C1008 cells were cultured for 12-18 hours (1.5 x 10^5^ ml) in 96-well cell culture plates to reach 70–80% confluence. An initial 1:10 dilution of samples (previously inactivated) was prepared with Dulbecco's modified Eagle’s medium (DMEM; Gibco) supplemented with 2% fetal bovine serum (FBS; ATCC) and 2% penicillin‒streptomycin-amphotericin B suspension (SIGMA). Twofold serial dilutions (1:10 to 1:5120) were prepared with 100 TCID_50_ of virus (vol:vol) and incubated at 37°C in 5% CO_2_ for 60 min. Then, 100 µL of this suspension was incubated with Vero cells, plus 100 µL of DMEM with 2% FBS and incubated at 37°C and 5% CO_2_ for 72 h. Samples were considered positive when a serum dilution of at least 1:10 neutralized the 100% cytopathic effect of the virus adjusted to 100 TCID_50_ (Bewley et al., 2021; Perera et al., 2020; Zhang et al., 2020).

ELISA

ELISA was performed as previously described (Melgoza-González et al., 2022). SARS-CoV-2 RBD and N protein (2 µg/mL) were used to coat Maxisorp ELISA microwell plates (Thermo Fisher Scientific) using 100 nM carbonate-bicarbonate buffer, pH 9.5, and incubated overnight (18-19 h) at 4°C. Then, plates were blocked (with a blocking buffer: 2% BSA (Sigma‒Aldrich), 3% glucose (Fagalab), and 0.025% sodium azide (Sigma‒Aldrich) for one hour at room temperature and washed three times with PBS/0.1% Tween 20 (PBST). Serum samples were diluted 1:100 in PBS with 0.05% Tween 20 and 1% nonfat milk (American Bio) and incubated for one hour at room temperature. After washing, anti-human IgG-HRP (Sigma‒Aldrich) was added and incubated for 30 min at room temperature, and after washing, 50 µL of 3,3',5,5'-tetramethylbenzidine (Immunochemistry, Minnesota USA) was added. The reaction was stopped with 50 µL of 1 M H_2_SO_4_, and the optical density (O.D.) was read at 450 nm using an automated spectrophotometer (Thermo Scientific Multiskan FC Microplate Photometer) during the next 5 min. The results were expressed as the relative arbitrary units (AU).

Statistical analysis

Statistical significance was determined by two-tailed Wilcoxon matched-pairs signed-ranks tests or Mann‒Whitney tests, and p values less than 0.05 were considered statistically significant. An unpaired t test was used to compare the anti-N and anti-RBD antibodies. All statistical tests were performed using GraphPad Prism, and p values less than 0.05 were considered statistically significant.

Figure. S1.

Anti-N IgG antibodies of samples from individuals at baseline. Indirect ELISA was performed to evaluate anti-N IgG antibodies. The number in the parentheses indicates the proportion of positive samples with nAbs above the detection limit. The dotted lines represent the cut-off. (-) negative samples, (+) positive samples.

Figure. S2.

Anti-RBD IgG antibodies in individuals at baseline and after booster with mRNA-1273. Indirect ELISA was performed to evaluate anti-RBD IgG antibodies. The values above the bars denote geometric mean titers, the number in the parentheses indicates the proportion of positive samples with nAbs above the detection limit. The dotted lines represent the cut-off.

Table S1.

Characteristics of individuals included in this study.

|  | n | (%) |
| --- | --- | --- |
| **Gender** |  |  |
| Female | 243 | 71.10 |
| Male | 94 | 27.89 |
| **Infection status** |  |  |
| Previous COVID-19 | 169 | 53.82 |
| Nonprevious COVID-19 | 145 | 46.17 |
| **Clinical manifestation** |  |  |
| Asymptomatic | 55 | 32.54 |
| Mild | 87 | 51.47 |
| Moderate | 22 | 13.01 |
| Severe | 5 | 2.95 |
| **Age** |  |  |
| 18 – 30 | 35 | 11.14 |
| 31 – 40 | 10 | 31.84 |
| 41 – 50 | 111 | 35.35 |
| 51 – 60 | 67 | 21.33 |
| >60 | 1 | 0.31 |
| **Health status** |  |  |
| Obesity | 56 | 33.13 |
| Diabetes | 23 | 13.60 |
| Smoking | 36 | 21.30 |
| Autoimmune disease | 12 | 7.10 |
| Cancer | 2 | 1.18 |
| Other | 40 | 23.66 |
|  |  |  |
